# Supplementary material for: FERN – a Java framework for stochastic simulation and evaluation of reaction networks
Source: BMC Bioinformatics. 2008 Aug 29;9:356. doi: 10.1186/1471-2105-9-356 (PMC2553347; doi:10.1186/1471-2105-9-356)
Supplement: Additional file 1 — FERN distribution, Version 1.3. This archive contains the FERN source code and binaries as well as documentation and example models in FernML and SBML. [file 1471-2105-9-356-S1.zip › fern/doc/javadoc/fern/tools/package-summary.html]

fern.tools


---


|  |  |  |
| --- | --- | --- |
| **Overview** \| **Package** \| Class \| **Use** \| **Tree** \| **Deprecated** \| **Index** \| **Help** | |  |
| **PREV PACKAGE**   **NEXT PACKAGE** | **FRAMES**    **NO FRAMES**     **All Classes** |  |


---

## Package fern.tools

Provides common used tools.

**See:**
  
          **Description**

| **Class Summary** | |
| --- | --- |
| **ColorSpectrum** | Provides methods to assign colors to numbers between 0 and 1. |
| **ConfigReader** | Reads simple config files for test cases and makes the data of it accessible. |
| **NetworkTools** | Contains various helper methods dealing with the `Network` interface. |
| **NumberTools** | Contains some common methods to handle number related data structures. |
| **Stochastics** | Manages a random number generators for various distributions. |

## Package fern.tools Description

Provides common used tools.

---


|  |  |  |
| --- | --- | --- |
| **Overview** \| **Package** \| Class \| **Use** \| **Tree** \| **Deprecated** \| **Index** \| **Help** | |  |
| **PREV PACKAGE**   **NEXT PACKAGE** | **FRAMES**    **NO FRAMES**     **All Classes** |  |


---
